# Metabolomics—A Promising Approach to Pituitary Adenomas

**DOI:** 10.3389/fendo.2018.00814

**Published:** 2019-01-17

**Authors:** Oana Pînzariu, Bogdan Georgescu, Carmen E. Georgescu

**Affiliations:** ^1^6^th^ Department of Medical Sciences, Department of Endocrinology, Iuliu Hatieganu University of Medicine and Pharmacy, Cluj-Napoca, Romania; ^2^Department of Ecology, Environmental Protection and Zoology, University of Agricultural Sciences and Veterinary Medicine Cluj-Napoca, Cluj-Napoca, Romania; ^3^Endocrinology Clinic, Cluj County Emergency Clinical Hospital, Cluj-Napoca, Romania

**Keywords:** pituitary adenoma, metabolomics, metabolite, mass spectrometry (MS), nuclear magnetic resonance (NMR), MALDI-MS, magnetic resonance spectroscopy (MRS)

## Abstract

**Background:** Metabolomics—the novel science that evaluates the multitude of low-molecular-weight metabolites in a biological system, provides new data on pathogenic mechanisms of diseases, including endocrine tumors. Although development of metabolomic profiling in pituitary disorders is at an early stage, it seems to be a promising approach in the near future in identifying specific disease biomarkers and understanding cellular signaling networks.

**Objectives:** To review the metabolomic profile and the contributions of metabolomics in pituitary adenomas (PA).

**Methods:** A systematic review was conducted via PubMed, Web of Science Core Collection and Scopus databases, summarizing studies that have described metabolomic aspects of PA.

**Results:** Liquid chromatography tandem mass spectrometry (LC-MS/MS) and nuclear magnetic resonance (NMR) spectrometry, which are traditional techniques employed in metabolomics, suggest amino acids metabolism appears to be primarily altered in PA. N-acetyl aspartate, choline-containing compounds and creatine appear as highly effective in differentiating PA from healthy tissue. Deoxycholic and 4-pyridoxic acids, 3-methyladipate, short chain fatty acids and glucose-6-phosphate unveil metabolite biomarkers in patients with Cushing's disease. Phosphoethanolamine, N-acetyl aspartate and myo-inositol are down regulated in prolactinoma, whereas aspartate, glutamate and glutamine are up regulated. Phosphoethanolamine, taurine, alanine, choline-containing compounds, homocysteine, and methionine were up regulated in unclassified PA across studies. Intraoperative use of ultra high mass resolution matrix-assisted laser desorption/ionization mass spectrometry imaging (MALDI-MSI), which allows localization and delineation between functional PA and healthy pituitary tissue, may contribute to achievement of complete tumor resection in addition to preservation of pituitary cell lines and vasopressin secretory cells, thus avoiding postoperative diabetes insipidus.

**Conclusion:** Implementation of ultra high performance metabolomics analysis techniques in the study of PA will significantly improve diagnosis and, potentially, the therapeutic approach, by identifying highly specific disease biomarkers in addition to novel molecular pathogenic mechanisms. Ultra high mass resolution MALDI-MSI emerges as a helpful clinical tool in the neurosurgical treatment of pituitary tumors. Therefore, metabolomics appears to be a science with a promising prospect in the sphere of PA, and a starting point in pituitary care.

## Introduction

Metabolomics, one of the newest “omics” sciences, assesses small molecules with molecular mass below 1,500 Da (1) within various bio-fluids (e.g., serum, plasma, cerebrospinal fluid, urine, saliva etc.) or tissues, to potentially set correlates to physiological or pathological status of an organism. Given its contribution to the understanding of cellular signaling mechanisms, in addition to identification and quantification of novel biomarkers in various clinical conditions, metabolomics is underpinning the development of personalized medicine.

Metabolites include carbohydrates, amino acids, nucleic acids, lipids, vitamins, organic acids, polyphenols, alkaloids, and inorganic species. A range of analytical techniques is applied, with either nuclear magnetic resonance (NMR), also known as magnetic resonance spectroscopy (MRS), or, more frequently, mass spectrometry (MS)-based platforms being routinely employed in assessing the metabolic fingerprint, the later method as a combination with other analysis techniques (i.e., gas-chromatography-mass spectrometry (GC-MS), liquid chromatography-mass spectrometry (LC-MS) (2, 3), ultra-performance liquid-chromatography tandem mass spectrometry (UPLC–MS/MS) (4, 5), ultra-high performance liquid chromatography-quadrupole time-of-flight mass spectrometry (UHPLC/Q*-*TOF*-*MS) (6, 7), capillary electrophoresis (CE-MS) (8, 9) or matrix-assisted laser desorption/ionization mass spectrometry (MALDI-MS) (10, 11) etc.) to overcome the limitations of MS, such as erroneous interpretation of the metabolomic analysis in presence of impurities or modest reproducibility of the method (12, 13). Further, complex informatics tools (e.g., principal component analysis, Mascot search etc.) that significantly improve identification of metabolomic panels by multivariate statistical analysis are integrated into most types of equipment.

Ionization of atoms and molecules followed by their separation according to the mass/charge ratio is a key principle of MS-based techniques; the methods most commonly used being electrospray ionization and electron impact ionization, followed by atmospheric pressure photoionization and atmospheric pressure chemical ionization (14).

Although sensitivity of MS is clearly superior to NMR spectrometry, the later is increasingly employed because the method is fast, highly reproducible and does not require additional steps of biological samples preparation, including separation and derivatization (15). In addition, NMR spectrometry can identify unknown compounds with identical masses, even those with different isotopic distribution.

The basic principle of NMR spectrometry consists of the spinning of atomic nuclei. The most common nuclei used in this technique are ^1^H (proton), ^13^P (phosphorus), ^15^N (nitrogen) and ^13^C (carbon), the highest sensitivity being attributed to ^1^H (16). With the time, MRS has proven to be a highly used and non-invasive technique in cerebral tumors, particularly *in vivo* MRS, using brain MRI images (17). The technique allows identification of various metabolites by obtaining signals from a cerebral region of interest (ROI), more exactly a three-dimensional volume of this region measuring at least 1 cm^3^ or the so-called *voxel* (18). However, a considerable disadvantage using this technique is the identification of a limited number of metabolites, those with extremely high concentrations (17).

Matrix-assisted laser desorption/ionization mass spectrometry imaging (MALDI-MSI) profiles as a valuable method that is able to identify peptides and proteins with a mass of up to 50,000 Da (19–21). In view of this aspect, MALDI-MSI appears to be useful in approaching pituitary gland disorders, since most of the pituitary hormones are proteins or peptides. The underlying principle of MALDI-MSI is to use a matrix that absorbs the energy emitted from an ultraviolet or infrared laser beam, followed by desorption and ionization of the analyzed metabolite, similarly to electrospray ionization (19). The method is fast and highly sensitive (22). Implementation of MALDI-MSI has made it possible to shift from assessing the metabolic fingerprint in biological products such as plasma, serum or urine directly to tissue sections.

A major advantage of the development of this technique is the correlation of MALDI-MSI results with the tumor histopathology, thus the demarcation of the tumor contour can be established. Further, MALDI-MSI is able to identify new biomarkers in an *in situ* context (i.e., paraphin embedded tissue or fixed tissue sections), while the combination of MALDI-MSI with computed tomography (CT), magnetic resonance imaging (MRI) or positron emission tomography (PET) imaging aims to improve the future approach to research (22).

Whichever technique is used, *untargeted* metabolomic analysis allows rapid and global description of a large number of metabolites (e.g., lipids, amino acids etc.), termed metabolomic fingerprint in a single sample that subsequently is subjected to interpretation and validation to define differences between physiological and pathological conditions (15, 23–25); while, *targeted* metabolomic analysis consists of the qualitative and quantitative assessment of a small number of preselected well known metabolites that are specific to a particular metabolic pathway (15).

The future prospects for improving the identification and quantification of metabolites are the combination of NMR spectrometry (MRS) and MS (26).

In past years, metabolomics has considerably developed in the field of endocrinology, including diabetes mellitus (27–30), obesity (27, 31, 32), polycystic ovary syndrome (33–37), thyroid cancer (38–41), osteoporosis (42, 43) and particularly adrenal diseases, i.e., adrenal cancer, Cushing's syndrome (44, 45), primary aldosteronism (46, 47) and pheochromocytoma (48) and resulted in description of novel cellular and molecular signaling mechanisms and characterization of complex panels of biomarkers of risk.

Metabolomics in pituitary disorders is currently at an early stage. In 2014, Höybye et al. (49) conducted a pilot study comparing serum metabolites in adult patients with growth hormone deficiency (GHD) to a healthy control group. The endpoint was to identify potential biomarkers for the diagnosis of GHD, concomitantly aiming to draw up a metabolomics-based individualized recombinant human GH (rhGH) replacement treatment protocol among affected subjects. Metabolomics analysis performed by GC-MS identified a number of 285 untargeted metabolites, 13 of them differentiating between patients with GHD and controls. Among these, lower levels of threonic acid, cystine, cysteine and palmitoleic acid and higher levels of glutamic acid, glyceric acid, aspartic acid, uridine and hypoxanthine-like were reported in adult GHD. Furthermore, rhGH treatment caused a decrease in levels of glutamic and glyceric acid and an increase in levels of hexadecanoic and palmitoleic acid.

Recently, Zhan and Desiderio (50) described the remarkable contribution that “omics” sciences, including metabolomics, will play in understanding the heterogeneity of pituitary adenomas (PA). The present review will focus on the contribution that metabolomics analysis techniques might provide to improve the diagnosis of PA, aiming to shed light on some molecular mechanisms underlying their tumor development.

## Materials and Methods

### Search Strategy and Eligibility Criteria

**Figure 1:**
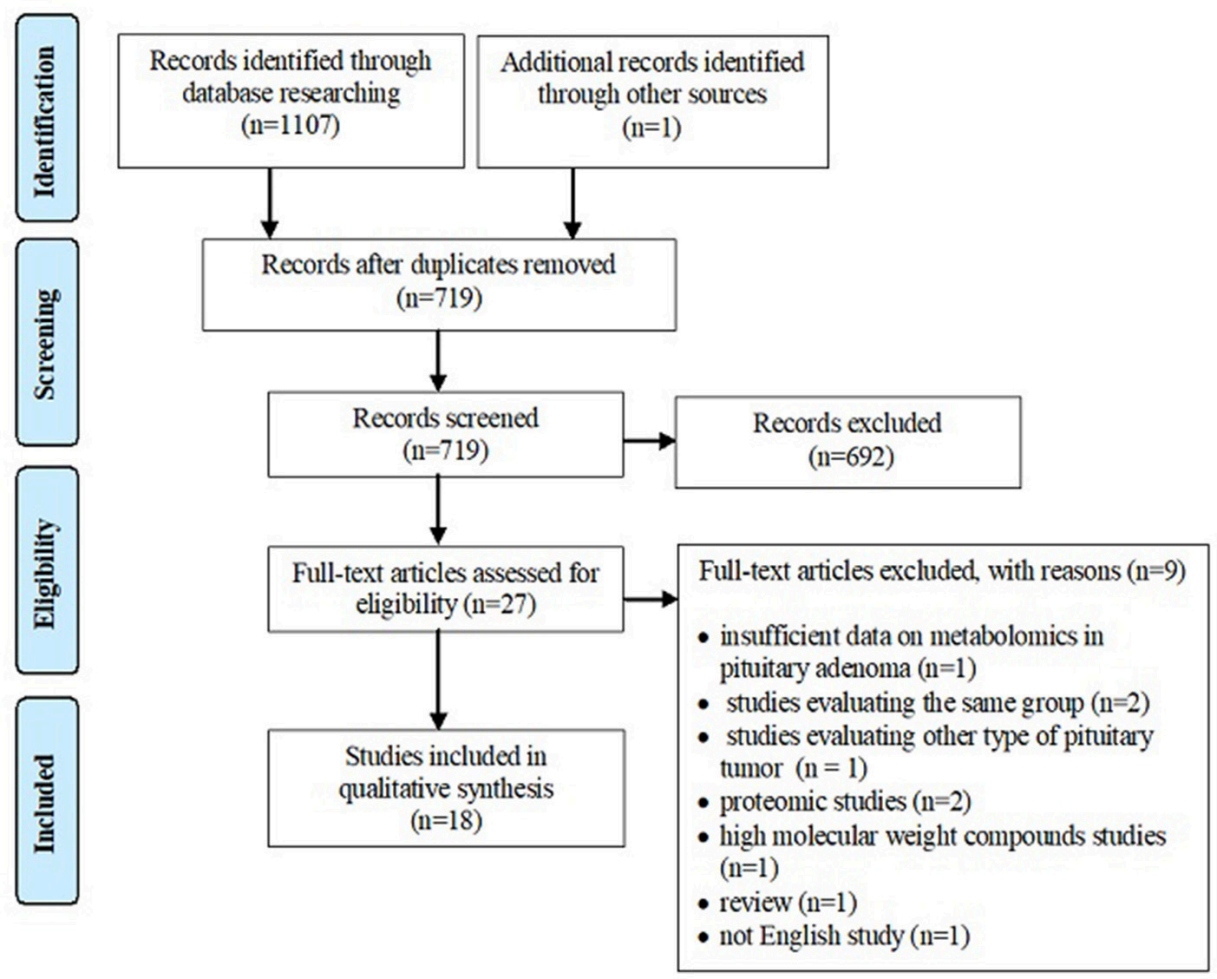
PRISMA style flowchart of the selected studies.

A systematic review of the literature was conducted independently by two of the authors via PubMed, Web of Science Core Collection and Scopus databases until 11th December 2018, using following keywords: *metabolomics pituitary adenoma/tumor, metabolomic biomarker pituitary adenoma/tumor, metabolomic analysis pituitary adenoma/tumor, metabolomic profile pituitary adenoma/tumor, metabolites pituitary adenoma, LC-MS pituitary adenoma/tumor, GC-MS pituitary adenoma/tumor, NMR spectrometry pituitary adenoma/tumor, MALDI pituitary adenoma/tumor, deoxycholic acid pituitary adenomas, 4-pyridoxic acid pituitary adenomas, phosphoethanolamine pituitary adenomas, alanine pituitary adenomas, N-acetyl aspartate pituitary adenomas, myo-inositol pituitary adenomas, 3-methyladipate pituitary adenomas, glutamate pituitary adenomas, glutamine pituitary adenomas, taurine pituitary adenomas*. Search keywords included specific metabolites to optimize data selection. The endpoint was to perform an overview of metabolomic aspects relevant to the approach of PA and potentially provide a source of detection and treatment targets for pituitary tumors. Inclusion criteria were represented by (1) studies that have evaluated the metabolomic profile or metabolomic biomarkers associated with PA or the contribution of NMR spectrometry and MS-based techniques in PA, (2) studies written in English, and (3) studies on human subjects. Exclusion criteria included (1) absence of PA group, (2) evaluation of other types of pituitary tumors, (3) studies that provide insufficient data on metabolomics in PA, (4) studies including overlapping groups of patients, (5) studies written in languages other than English, (6) proteomics studies, (7) high molecular weight compounds studies, and (8) reviews. According to the flowchart (Figure 1), 1,107 articles were included for analysis by searching electronic databases. Additionally, an article has been added to our research through hand searching (51). Duplicate (*n* = 389) and irrelevant (*n* = 692) articles were excluded. An article was excluded due to insufficient data on metabolomics in PA (52). Three articles studied the same group of patients with PA (53–55), so only the last one (55) was included in our review, the other two being excluded (53, 54). An article was excluded because it was not written in English (56). Two articles were excluded because they provided proteomics data (57, 58) and another article was excluded, evaluating compounds with a molecular weight greater than 1,500 Da (59). A study that evaluated other types of pituitary tumors (60) and a review (61) were also excluded. Finally, 18 studies enrolling 241 patients with PA were eligible for our review (Table 1). Of these, 8 articles described *in vivo* MRS approach in PA, providing specific metabolites measurements.

**Table 1:** Clinical cases of pituitary adenomas (PA) included for metabolomics analysis.

**No. of cases**	**Pituitary adenoma type**	**No**.	**Percentage (%)**
241 patients	ACTH-secreting pituitary adenoma	26	10.78
	GH-secreting pituitary adenoma	8	3.31
	PRL-secreting pituitary adenoma	36	14.93
	GH and PRL-secreting pituitary adenoma	4	1.65
	FSH-secreting pituitary adenoma	2	0.82
	FSH and LH-secreting pituitary adenoma	8	3.31
	Clinically non-functional pituitary adenoma	28	11.61
	Pituitary adenoma (functional and non-functional)	129	53.52

### Study Quality Assessment

Assessment of the quality of included studies was performed using the QUADAS2 tool (62) following four domains: patient selection, index test, reference standard and flow and timing. The index test was represented by metabolomic analysis. Histopatological examination was the reference standard for PA diagnosis. We evaluated the risk of bias using all four domains of QUADAS2 tool. We also used the first three domains for the concern regarding applicability.

## Results

### Metabolomics vs. Immunohistochemistry in Functional PA

Calligaris et al. (63) demonstrated the specific endocrine functionality of both non-pathological pituitary tissue (6 samples) and pituitary tumors (45 samples), using MALDI-MSI. The purpose of this study was to locate functional PA and to identify the delineation between them and healthy pituitary tissue, an aspect of potentially key relevance, especially during surgery, on one hand facilitating total adenoma resection and on the other hand preserving vasopressin secretory cells within the neurohypophysis and pituitary stalk, thus avoiding central diabetes insipidus, the most common postoperative complication. In a first step, using positive*-*mode MALDI*-*MS, the metabolic fingerprint of non-pathological pituitary tissue was assessed, to confirm presence of vasopressin and neurophysin 2 in the neurohypophyseal tissue in addition to GH, α-melanocytic stimulation hormone (MSH), adrenocorticotrophic hormone (ACTH), β-endorphin, joining peptide, γ-lipotrophin (LPH), and β-LPH in the anterior pituitary (Table 2). Afterwards, using matrix sublimation/recrystallization, prolactin (PRL) was identified in the lactotrophic area of the anterior lobe, in addition to detection of neurophysin 1 in the posterior pituitary. The distribution of these metabolites, reported by this study, highly corresponds to the immunohistochemical distribution of hormones within the pituitary gland (79).

**Table 2:** Metabolomic aspects in pituitary adenomas (PA).

**No**.	**Reference**	**Biological sample**	**Analytical Technique**	**Results**
1.	Calligaris et al. (63)	45 PA vs. 6 healthy pituitary glands	MALDI-MSI	**Non-pathological pituitary tissue** ▸ presence of vasopressin, neurophysin 2 and neurophysin 1 in the neurohypophysis ▸ identification of GH, α-MSH, ACTH, β-endorphin, joining peptide, γ-LPH, β-LPH and PRL in the anterior lobe ▸ identification of b- and y-series ions of vasopressin and c-series ion fragments of GH (c13), ACTH (c10) and neurophysin 2 (c16) in the posterior and anterior pituitary gland, respectively**Pituitary adenomas** ▸ elevated levels of GH, PRL and ACTH have been identified within GH-secreting PA, PRL-secreting PA and ACTH-secreting PA, respectively ▸ identification of the demarcation between PA and healthy pituitary tissue
2.	Oklu et al. (64)	16 central blood samples (plasma) from 7 patients with ACTH-secreting PA, who underwent bilateral IPSS vs. 9 control samples	LC-MS/MS	**Metabolites:** ▸ deoxycholic acid, 3-methyladipate, pyridoxate **Pathways:** ▸ alanine, aspartate and glutamate metabolism (the most affected), ▸ vitamin B metabolism, ▸ lysine biosynthesis, ▸ purine metabolism, ▸ amino sugar and nucleotide sugar metabolism, ▸ glycolysis and gluconeogenesis, ▸ aminoacetyl-tRNA biosynthesis, ▸ starch and sucrose metabolism
3.	Feng et al. (65)	brain tissue samples from 6 PA with ACTH-secreting PA vs. 7 healthy brain samples	GC-MS	**Metabolites:** up-regulation of short chain fatty acids (heptanoic acid, octanoic acid, nonanoic acid, hexanoic acid and capric acid), down-regulation of glucose-6-phosphate **Pathways:** ▸ fatty acids metabolism, ▸ glycolysis/gluconeogenesis
4.	Ijare et al. (51)	post-surgery tumor tissue samples from 3 gonadotropin-secreting PA and 3 PRL-secreting PA	NMR spectrometry	**Metabolites:** ▸ phosphoethanolamine, glutamate, glutamine, N-acetyl aspartate, aspartate and myo-inositol - significantly altered in both types of adenomas ▸ down regulation of phosphoethanolamine, N-acetyl aspartate and myo-inositol and up regulation of aspartate, glutamate and glutamine in PRL-secreting PA compared to gonadotropin-secreting PA
5.	Lee et al. (66)	urine samples from 27 PRL-secreting PA *vs*. 31 healthy group	GC-MS	**Metabolites:** up regulation of estrogen metabolites and 17-ketosteroids, high level of c5 beta/5 alpha-hydrogensteroids and delta 5/delta 4-steroids ratio
6.	Kinoshita et al. (55)	2 surgically excised samples of PA vs. 4 non-tumorous brain samples	^1^H-MRS	**Metabolites:** up regulation of phosphoethanolamine, taurine and alanine
7.	Jarmusch et al. (67)	brain tissue sample from 14 PA *vs*. normal brain parenchyma and other brain tumors (gliomas, meningiomas)	DESI-MS	**Metabolites:** lipids
8.	Bicíková et al. (68)	brain tissue sample from 25 patients with PA vs. meningioma, glioma and glioblastoma	GC/FID	**Metabolites**: up-regulation of homocysteine and methionine
9.	Usenius et al. (69)	brain tissue sample from 6 PA vs. normal brain tissue	^1^H-MRS	**Metabolites:** down-regulation of N-acetyl aspartate and creatine and up regulation of choline-containing compounds
10.	Solivera et al. (70)	brain tissue sample from 3 PA vs. 3 health brain tissue	^31^P-MRS	**Metabolites:** up regulation of phosphatidylinositol and phosphatidylcholine, down regulation of phosphatidylserine and sphingomyelin
11.	Einstein et al. (71)	brain MRI sequences from 28 PA	^1^H-MRS	**Metabolites:** down-regulation of N-acetyl aspartate and creatine and up regulation of choline-containing compounds
12.	Chernov et al. (72)	brain MRI sequences from 19 PA	^1^H-MRS	**Metabolites:** down-regulation of N-acetyl aspartate and creatine and up regulation of choline-containing compounds
13.	Faghih Jouibari et al. (73)	brain MRI sequences from 10 non-functional PA	^1^H-MRS	**Metabolites:** up regulation of choline
14.	Isobe et al. (74)	brain MRI sequence from 5 PA vs. 7 healthy volunteers	^1^H-MRS	**Metabolites:** up regulation of choline, lack of N-acetyl aspartate and total creatine
15.	Sutton et al. (75)	brain MRI sequences from 3 non-functional PA *vs*. 17 healthy individuals	^1^H-MRS	**Metabolites:** up regulation of choline
16.	Stadlbauer et al. (76)	MRI sequences from 27 functional and non-functional PA	^1^H-MRS	**Metabolites:** up regulation of choline
17.	Kozić et al. (77)	brain MRI sequences from 1 GH-secreting PA	^1^H-MRS	**Metabolites:** up regulation of choline
18.	Khiat et al. (78)	brain MRI sequences from 7 ACTH-secreting PA vs. 40 healthy individuals	^1^H-MRS	**Metabolites:** down regulation of choline

*PA, pituitary adenoma; MALDI-MSI, matrix-assisted laser desorption/ionization mass spectrometry imaging; LC-MS/MS, liquid chromatography tandem mass spectrometry; GC-MS, gas chromatography-mass spectrometry; ^1^H-MRS, proton magnetic resonance spectroscopy; NMR, nuclear magnetic resonance; DESI-MS, desorption electrospray ionization-mass spectrometry; GC/FID, gas chromatography with flame ionization detection; ^31^P-MRS, ^31^P-magnetic resonance spectroscopy*.

GH, ACTH and neurophysin 2 were identified by Mascot searches. In addition, secretion of these peptides was confirmed by MALDI in-source decay (ISD) fragmentation, by identification of c-series ion fragments of GH (c13), ACTH (c10) and neurophysin 2 (c16) in the anterior and posterior pituitary gland. Moreover, the identification of b- and y-series ions of vasopressin was possible using MALDI time-of-flight/time-of-flight mass spectrometry (MALDI TOF/TOF).

In his study, Calligaris et al. (63) included ACTH- (*n* = 6), GH- (*n* = 7), PRL- (*n* = 6), GH- and PRL- (*n* = 4), FSH- (*n* = 2), FSH- and LH-secreting PA (*n* = 5) and clinically non-functional PA (*n* = 15). In the majority of cases, metabolomics analysis confirmed hormonal hypersecretion within pituitary tumor cells in agreement to clinical and biochemical suspicion. Metabolomics data correlated with histopathological findings, including haematoxylin-eosin and reticulin staining, respectively. Therefore, identification of intact GH and the c13 ion fragment, corroborated with a disruption of reticulin fiber network diagnosed GH-secreting PA. In the same manner, identification of intact ACTH and the c10 ion fragment, corroborated with a disruption of reticulin fiber network diagnosed ACTH-secreting PA.

Besides the ability to confirm pituitary hypersecretion, MALDI-MSI seems to be a good approach in differentiating pituitary tumor tissue from intact pituitary tissue with an overall specificity (Sp) of 93% and a sensitivity (Se) of 83%. To be emphasized, sensitivity was highly variable among various types of PA, reaching 100% in ACTH-secreting PA but only 50% in prolactinomas and 82% in GH-secreting PA. On the contrary, specificity was high, irrespective of the type of PA (i.e., 93% in ACTH-secreting PA and 100% in PRL- and GH-secreting PA, respectively).

### Metabolomic Pathways in ACTH-Secreting PA

In a metabolomics research, Oklu et al. (64) evaluated 8 patients with suspicion of Cushing's disease in whom clinical features of hypercorticism were present (i.e., weight gain, hypertension, osteoporosis, easy bruising, moon face, fatigue, diabetes mellitus, and hirsutism), nonetheless with indeterminate pituitary imaging.

To confirm diagnosis, all patients underwent bilateral inferior petrosal sinus sampling (IPSS). ACTH-secreting PA was confirmed in 7 patients, while in one patient the diagnosis was excluded. The metabolic profile was determined in plasma samples from the ipsilateral IPS of patients (7 samples) and compared to contralateral samples plus two samples from the patient in whom ACTH hypersecretion failed to be confirmed (9 samples).

Postoperative follow-up showed improvement of symptomatology in 4 patients and disease remission in the remaining three.

Using LC-MS/MS, 12 distinct metabolites were reported in patients with ACTH-secreting PA in comparison to the control group, specifically 2-hydroxybutyric acid, aminoadipic acid, L-aspartic acid, 3-hydroxyphenylacetic acid, hypoxanthine, 4-pyridoxic acid, quinolinic acid, sucrose, xanthine, glucose 6-phosphate, deoxycholic acid, and 3-methyladipate. After Bonferroni adjustment, however, only deoxycholic and 4-pyridoxic acids and 3-methyladipate remained statistically significant (Table 2).

Using Kyoto Encyclopedia of Genes and Genomes (KEGG) pathway database, 8 main pathways affected in Cushing's disease were identified (64) that involved: (1) Alanine, aspartate and glutamate metabolism, which appeared to be the most affected metabolic pathway, (2) Vitamin B metabolism, (3) Lysine biosynthesis, (4) Purine metabolism, (5) Amino sugar and nucleotide sugar metabolic pathways, (6) Glycolysis and gluconeogenesis pathways, (7) Aminoacetyl-tRNA biosynthesis, and (8) Starch and sucrose metabolism (Table 2).

Recently, Feng et al. (65) conducted a metabolomic (via GC-MS) and proteomic study in a group of patients with ACTH-secreting PA. For metabolomic analysis, the author included brain tumor samples from 6 patients with ACTH-secreting PA vs. healthy brain tissue from 7 control subjects. It was found that short chain fatty acids (heptanoic acid, octanoic acid, nonanoic acid, hexanoic acid and capric acid) were up regulated, while glucose-6-phosphate was down regulated. Thus the metabolomic pathways involved in PA were the metabolism of fatty acids and glycolysis/gluconeogenesis (Table 2).

### Metabolomics Studies in Gonadotropin- and PRL-Secreting PA

In 2017, Ijare et al. (51) used *ex vivo* NMR spectrometry to assess the metabolomic profile of postoperative pituitary tissue sampled from patients with gonadotropin- and PRL-secreting PA, respectively, with the main finding that both types of PA contain central nervous system metabolites such as phosphoethanolamine, glutamate, glutamine, N-acetyl aspartate, aspartate, and myo-inositol.

When comparing these two types of PA, it was found that phosphoethanolamine, N-acetyl aspartate and myo-inositol are down regulated in prolactinoma, whereas aspartate, glutamate and glutamine are up regulated (Table 2). Ijare's study is currently underway, so a larger number of patients could provide additional insights into the evaluation of the metabolomic fingerprint in these types of pituitary tumors. However, a true control group was apparently not considered, a major limitation of this study.

Lee et al. (66) conducted a study of 26 women with PRL-secreting PA compared to 31 healthy controls, analyzing their urine using GC-MS. A high level of all estrogen metabolites and 17-ketosteroids in the urine of these patients was shown. In addition, high c5 beta/5 alpha-hydrogensteroids and delta 5/delta 4-steroids ratios were identified (Table 2).

### Metabolites of PA Compared to Other Brain Tumors

In 2015, Jarmusch et al. (67) performed a study of 58 brain tumors, including 14 patients with PA, the rest of the tumors being gliomas, astrocytomas and meningiomas. Analyzing brain tissue samples through desorption electrospray ionization (DESI)-MS, the author identified lipid peaks, which allow differentiation of PA from normal brain parenchyma and other brain tumors (gliomas, meningioma), respectively (Table 2). Moreover, the discriminant model of brain tumors using DESI-MS shows an overall Sp of 99.7% and Se of 99.4%. The metabolomic profile was explicitly described in the case of gliomas, indicating a decrease in N-acetyl aspartate and 2-hydroxyglutaric acid, while this was not very well achieved in PA.

Bicíková et al. (68) analyzed tumoral brain tissue samples from 25 patients with PA within a series of brain tumors, using GC with flame ionization detection (GC/FID). Patients with PA presented a marked increase in homocysteine. Homocysteine was also increased in patients with glioblastoma. At the opposite pole, meningiomas and gliomas were characterized by low level of homocysteine. Likewise, PA were characterized by an increased level of methionine, while gliomas exhibited low levels of this amino acid (Table 2).

A large body of evidence resulted from MRS-based studies (55, 69, 70) to show alterations of phospholipid metabolism in PA tumor samples as evidenced by high levels of phosphoetanolamine (55), phosphatidylcholine (69, 70) and phosphatidylinositol (70) concentrations (Table 2). The pattern is not specific, as phosphoetanolamine and choline-containing compounds were abundantly present in meningioma (55), medulloblastoma (80), glioblastoma (81) and malignant lymphoma tumor samples (70). Cerebral metastases from hepatocellular carcinoma presented high concentrations of choline-containing compounds, while craniopharyngiomas showed decreased levels of these (55). Elevated alanine but low N-acetyl-aspartate concentrations were reported in PA, nonetheless, a similar pattern was apparent in meningioma and gliomas (55, 69). Additionally, a high level of taurine was observed in PA, medulloblastoma and cerebral metastases with kidney starting point (55, 80) (Table 2). Ependymoma presented an increased level of myo-inositol, whereas pilocytic astrocytoma exhibited increased levels of fatty acids (80).

An increased concentration of glycine was linked to neuroectodermal tumors (81), while neurinomas, glioblastomas (55) and ependymomas (80) showed a high peak of myo-inositol.

### PA Metabolites by *in vivo* Proton MRS

A series of studies performed single vortex proton (^1^H)-MRS on patients with various suprasellar tumors that included cases of PA. Across 3 studies including a total of 57 patients with both functional (29/57) and non-functional (28/57) PA, markedly decreased N-acetyl aspartate levels were demonstrated by single vortex ^1^H MRS, in addition to absent or low levels of creatine and moderately elevated levels of choline-containing compounds (71–73) (Table 2). Moreover, in up to 50% of cases, including 2 cases of pituitary apoplexy, the concentration of N-acetyl aspartate remained unidentified. No significant differences between the two types of PA were observed (71, 72). Nonetheless, a similar metabolomic pattern was found in suprasellar gliomas and chordomas while craniopharyngiomas presented low levels of all evaluated metabolites (71, 72). Further, it was found that in diagnosis of suprasellar tumors the overall efficacy of proton MRS in association with MRI (87%) was greater than MRI alone (69.6%), but this difference was not statistically significant (73). Referring to other types of suprasellar tumors, the authors reported low levels of N-acetyl aspartate and creatine and high levels of choline-containing compounds in the case of gliomas. Chordomas showed low levels of N-acetyl aspartate and creatine, but high levels of lipids and choline-containing compounds. Craniopharyngiomas showed low levels of all evaluated metabolites (71, 72).

Likewise, in a series of brain tumors from 57 patients, including 5 PA vs. 7 healthy volunteers, Isobe et al. (74) identified an increased peak of choline and a lack of N-acetyl aspartate and total creatine. The choline peak was confirmed in a small series of 3 children with non-functional PA (75) (Table 2).

Stadlbauer et al. (76) evaluated 27/37 patients with large functional and non-functional PA and a volume ≥4 cm^3^. Of the 27, 11 PA presented hemorrhagic areas, while 16 were non-hemorrhagic. Non-hemorrhagic PA group revealed a peak of choline (Table 2). The concentration of this metabolite was strongly correlated with the MIB-1 index on the immunohistochemical examination of these 16 patients.

Kozić et al. (77) described the case of a 41-year-old patient with an ectopic 53/40 mm pituitary macroadenoma. Preoperatively, the patient was examined using *in vivo* single vortex ^1^H-MRS, and a high peak of choline was noticed (Table 2). Due to disease persistence, after surgery the patient required treatment with somatostatin analogs (lanreotide 120 mg/4 weeks). Approximately 1 year later marked adenoma shrinkage and the lack of choline peak in the tumor were demonstrated along with a favorable clinical outcome.

Khiat et al. (78) evaluated a series of 13 patients including 7 patients with ACTH-secreting PA and 6 patients with ACTH-independent Cushing's syndrome vs. 40 healthy individuals. The objective of the study was the characterization of cerebral metabolites in the thalamic, temporal and frontal region by analyzing the brain MRI sequences. Irrespective of the etiology of Cushing's syndrome, choline/creatine ratio showed a marked decrease in the thalamus and frontal area (Table 2).

### Quality Assessment of Studies

**Figure 2:**
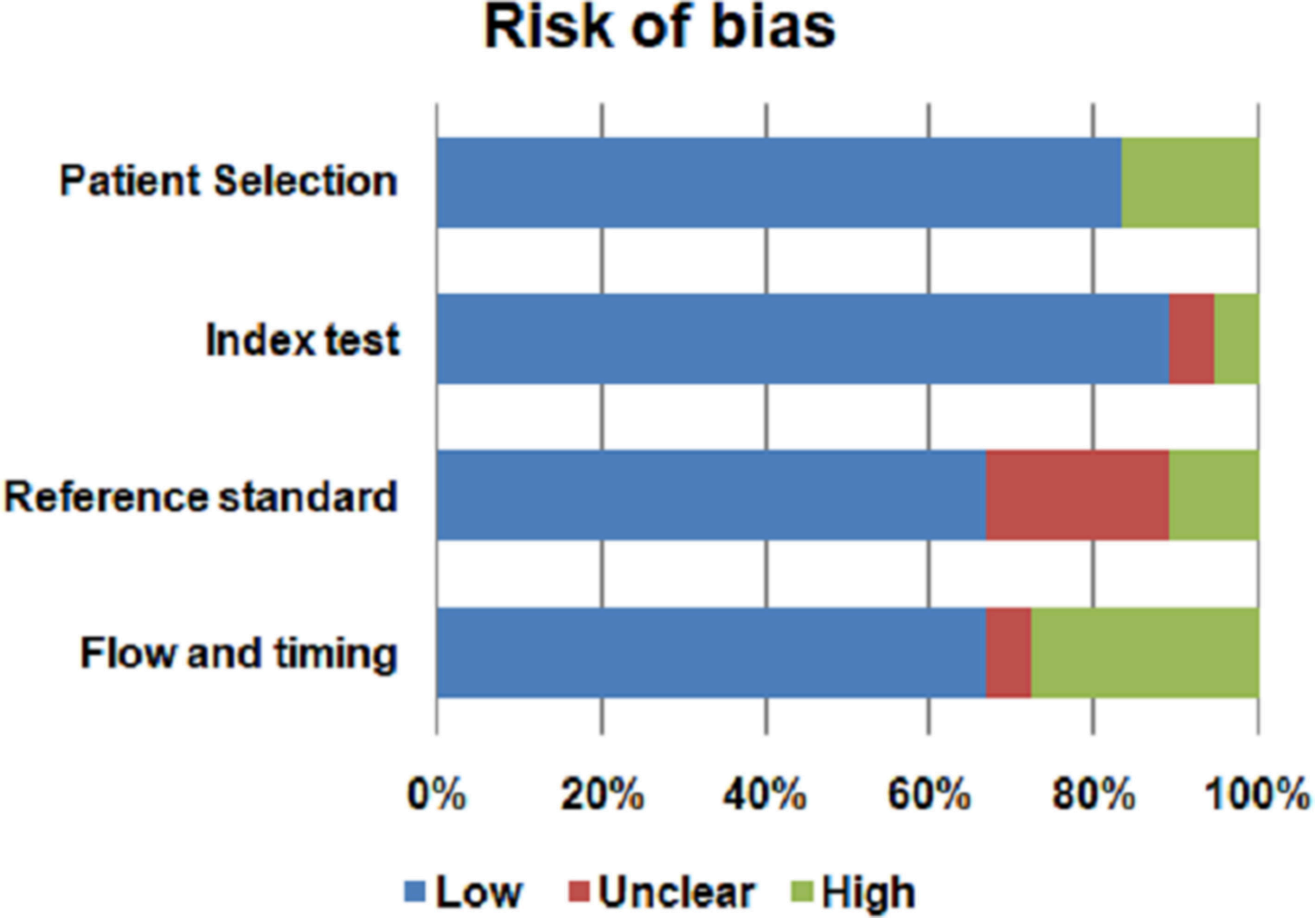
Risk of bias of the included studies.

**Figure 3:**
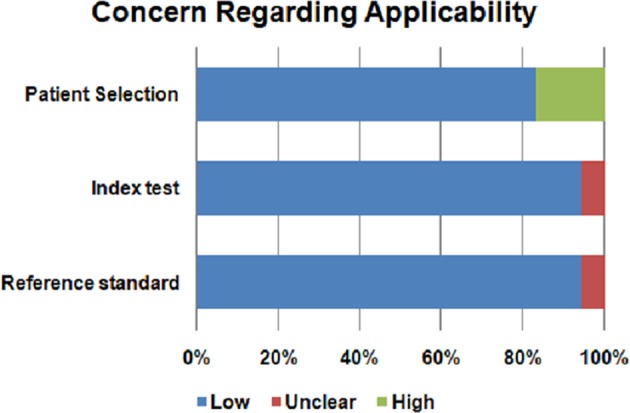
Concern regarding applicability of the included studies.

The risk of bias and the concern regarding applicability in included studies is illustrated in Figures 2, 3. Patient selection in studies was low in 16.66% of cases. Regarding the index test, 88.88% of studies accurately described the metabolomic analysis. The reference standard for PA diagnosis was histopatological examination that was described in 66.66% of cases. The flow of patients through the study and timing of reference standard and the index test were low in 27.77% of cases.

## Discussion

The approach of metabolomics techniques in neuroendocrinology and neurosurgery has grown in recent years, providing additional information to genomics and proteomics. As from the metabolomic perspective, glioblastoma, the most common and severe type of brain cancer in adults (82), has begun to be studied years ago when elevated concentration of phosphocholine was reported and found to be even higher in primary glioblastoma compared to recurrent disease (81). Recently, MALDI-MSI has been successfully implemented in differentiation of brain tumor vs. healthy brain tissue on a murine model of high-grade glioblastoma (83).

Metabolomic analysis has already proven its potential with regard to brain tumors, as in the classification of both meningioma and astrocytoma, depending on their aggressiveness. High-grade meningioma presents decreased levels of alanine and creatine in comparison to low-grade meningioma (84). Also, N-acetyl aspartate, myo-inositol, lactate, creatine and glycine show statistically significant differences depending on tumor aggression in astrocytoma (85).

**Figure 4:**
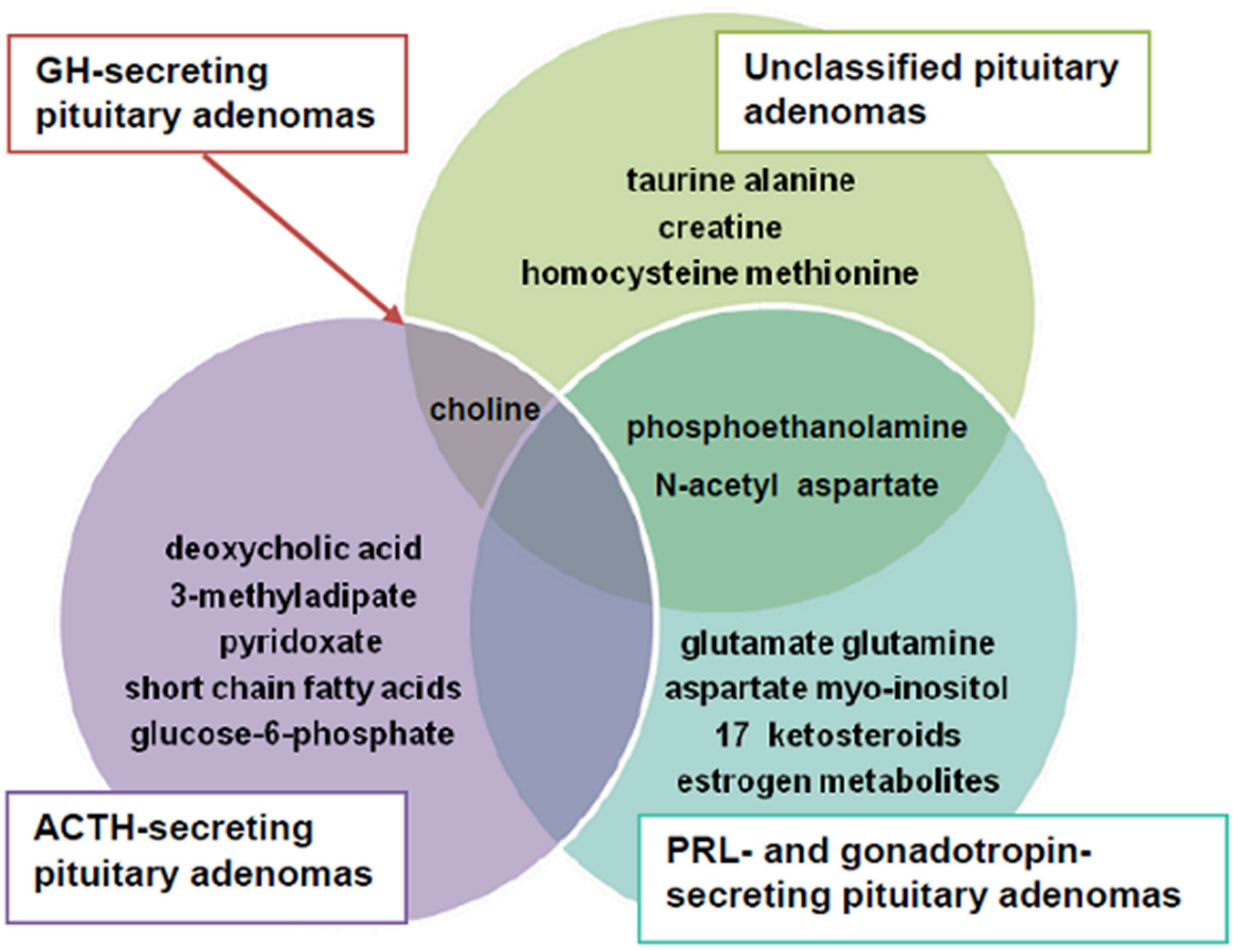
The main altered metabolites in pituitary adenomas (PA).

The present review, conducted in a systematic manner, identified a series of metabolites to be altered in PA as illustrated in Figure 4.

Summarizing data, we observed that N-acetyl aspartate, choline-containing compounds and creatine were main metabolites highly effective in differentiating PA from healthy tissue. The most obvious pattern consisted of decreased N-acetyl aspartate and creatine and increased choline-containing compounds levels.

N-acetyl aspartate is a derivative of aspartic acid abundantly found in the human brain. Except for Canavan disease, a genetic disorder characterized by toxic accumulation of N-acetyl aspartate caused by aspartoacylase inactivation (86), down regulation of this metabolite seems to be constant in clinical conditions that associate neuronal dysfunction, including epilepsy (87–89), multiple sclerosis (90), Alzheimer's disease (91, 92), schizophrenia (93, 94), stroke (95), brain injury (96, 97) and brain tumors (98, 99).

Choline-containing compounds (choline, phosphatidylcholine etc.) are involved in the synthesis and degradation of the cell membrane. Thus, a high concentration of these compounds is suggestive of accelerated cell membrane turnover in PA (100). In addition, the high level of choline correlates with tumor proliferation (71). Decreased creatine availability may suggest a disruption of energy metabolism in PA, given that this metabolite plays an important role in the ATP/ADP cycle (71).

Phosphoethanolamine, which appeared to be the key metabolite found in pituitary tumor tissue vs. non-tumoral pituitaries, is a precursor of phosphatidylcholine and phosphatidylethanolamine, both of which are components of cell membranes. It seems that phosphoethanolamine down regulation is involved in tumor genesis, given that pharmacologic administration of phosphoethanolamine suppressed tumor growth both *in vivo* and *in vitro* studies performed in mice bearing melanoma (101). In line with that, Ferreira et al. (102) reported that synthetic phosphoethanolamine reduces tumor growth and has an inhibitory action on clonal metastases in an acute promyelocytic leukemia model.

Another observation of our review included the alteration of myo-inositol, aspartate, glutamate and glutamine in gonadotropin- and PRL-secreting PA. More than that, phosphoethanolamine, myo-inositol and N-acetyl aspartate were down regulated in prolactinoma, whereas aspartate, glutamate and glutamine were up regulated.

Myo-inositol, a component of many phospholipids that is found in considerable amounts in the brain functions as a second messenger in multiple intracellular signaling pathways. The low concentration of myo-inositol is linked to an imbalance in osmolyte function of malignant cells (103, 104). Myo-inositol is responsible for cell cycle control, apoptosis, inhibition of the PI3K/Akt pathway and NF-kB activity (105). Moreover, it exerts antioxidant, anti-inflammatory and anti-tumoral effects, through insulin modulation (104). Its potential role has been described in breast and colon cancer (106, 107). Accordingly, Kesler et al. (106) reported an increased level of prefrontal myo-inositol in a group of 19 breast cancer survivors after chemotherapy. Moreover, Derbal-Wolfrom et al. (107) postulated that myo-inositol trispyrophosphate treatment increased the oxygen load to result in inhibition of colon tumor growth and stimulation of homeobox gene Cdx2 expression within the intestinal wall.

As in the research conducted by Oklu et al. (64), Ijare et al. (51) also identified that the aspartate and glutamate metabolism is affected in PRL-secreting PA. Glutamine is another abundant free amino acid with roles in protein synthesis. Thus, it plays important roles in the growth of normal and cancerous cells and many studies have demonstrated these cells are dependent on glutamine concentration (108, 109), while glutamine deprivation on cell cultures is associated with malignant cells death (110, 111). The down regulation of this metabolite, which is particularly emphasized in PRL-secreting PA, seems to be involved in tumor genesis. At the opposite pole, the up-regulation of phosphoethanolamine in unclassified PA may suggest an activation of phosphatidylethanolamine metabolism, which is involved in membrane shape changes in tumor cells (55).

Alterations of the amino acids metabolism in PA, especially involving alanine, glutamate and aspartate represents another feature that deserves to be taken into account. Alanine, a non-essential amino acid, is an end product of glutamate oxidation, which is a major source of respiratory energy in the tumor cell. In addition, alanine exerts proliferative effects on malignant cells (112, 113).

A similar effect is attributed to glutamate (114); its actions are mediated through two main receptors predominantly expressed in the brain: ionotropic glutamate receptors (iGluRs) and metabotropic glutamate receptors (mGluRs) (114). Recent studies have described these receptors in peripheral organs and implicitly in a wide range of cancers such as lung, thyroid, digestive tract, breast and ovarian cancer (115–120).

Aspartate is a metabolite of the urea cycle, involved in gluconeogenesis, being derived from asparaginase. The concentration of this metabolite is decreased in malignant cells, being synthesized from oxaloacetate by aminotransferase activity (121). In 2015, Xie et al. (122) noticed a decreased level of aspartate in the plasma of 35 patients with breast cancer. In addition, Dornfeld et al. (123) demonstrated improved cell mitochondrial function and reduction of doxorubicin toxicity in breast cancer samples.

Up-regulation of homocysteine in PA may be responsible for citotoxicity (68) and oxidative stress. Increased levels of homocysteine have also been reported in anxiety disorders, Alzheimer's disease, dementia, breast cancer and thyropathies (68). Moreover, the alteration of methionine metabolism leads to neurological dysfunction (68).

The identification of estrogen metabolites and 17-ketosteroids in the urine of patients with PRL-secreting adenomas is most likely to be due to decreased activity of 3 beta-hydroxysteroid dehydrogenase and 5α-reductase that occurs among these patients (66).

Another important finding is the identification of deoxycholic acid, 4-pyridoxic acid, 3-methyladipate, short chain fatty acids and glucose-6-phosphate as potential metabolomic biomarkers in patients with Cushing's disease (64, 65).

Deoxycholic acid is a bile acid that acts as a fat emulsifier, improving intestinal absorption. Moreover, it induces deoxyribonucleic acid (DNA) damage by increasing intracellular production of oxidative stress (124, 125). Up to now, increased levels of deoxycholic acid have been demonstrated in digestive (126, 127) and breast cancers (128), but its involvement in the appearance of pituitary tumors has not yet been documented.

4-pyridoxic acid is a urinary catabolite of vitamin B6, being a biomarker of vitamin B6 status. Thus, deficiency of 4-pyridoxic acid is correlated with a deficiency in vitamin B6 that has been shown to be involved in tumor development and its progression by following mechanisms: cell cycle alteration, angiogenesis, chromosomal instability, inflammation and increased oxidative stress (129–131).

13-methyladipate is a catabolic product of phytanic acid, being a saturated fatty acid obtained by eating dairy products and ruminant meat. Recent studies have shown that the intake of phytanic acid is associated with an enhanced risk of non-Hodgkin lymphoma (132) and prostate cancer (133, 134).

Metabolic pathway analysis has shown that amino acid metabolism is the most altered one among patients with Cushing's disease. The same aspect has been highlighted in other endocrine tumors, such as thyroid cancer, where an elevated concentration of methionine, glutamine, glycine, tyrosine and taurine was reported (39, 103, 104, 135–139). This high level of amino acids is linked to cell proliferation and energy substrate. The main sources of amino acids are represented by the combination between the increase of protein catabolism, *de novo* amino acids synthesis and augmented extracellular matrix degradation (140–142). On the other hand, by using ^1^H-NMR, a down-regulation of leucine, lysine, valine, serine, alanine and tyrosine was observed in plasma and serum of patients with thyroid cancer (138, 139). Also, alterations in amino acid metabolism have been observed in ovarian (143), breast (144) and prostate cancer (145)

Another important aspect was the involvement of short chain fatty acids metabolism in the pathogenesis of ACTH-secreting PA. This dysregulation is likely correlated with the increased cell turnover and lipids demand in membrane biosynthesis needed for cell proliferation (146).

Likewise, the decreased level of glucose-6-phosphate, which is a glycolic metabolite, along with pyruvate, represents a first step in demonstrating the involvement of glycolysis/gluconeogenesis pathway in ACTH-secreting PA.

The contribution of MALDI-MSI technique in the diagnosis of functional PA appears to be a major one, due to its high sensitivity and specificity in identifying pituitary hormones and fragments in addition to the possibility of delineating pituitary tumor tissue, making this method the most pertinent metabolomic assessment to be employed in the investigation of pituitary tumors.

The present review shows several limitations. Although conducted in a systematic fashion, only few articles could be included. Further, the limited number of patients/samples included in studies, the different types of biological samples analyzed (plasma, post-surgical tumor tissue, urine), differences in analytical techniques and a lack of homogeneity of PA may explain the heterogeneous results. Sensitivity and specificity of the metabolomic techniques was described in few studies. Moreover, the use of single voxel ^1^H-MRS in some studies was able to identify only a limited number of metabolites by analyzing the brain MRI sequences. Furthermore, the sensitivity of these methods in the detection of PA needs to be considerably improved, perhaps the best approach in the future would imply the combination of NMR and MS-based techniques, the implementation of MALDI-MSI on an increased group of patients or the combination of MALDI-MSI with CT, MRI or PET imaging.

### Future Perspectives

Although an emerging science, metabolomics has a huge potential compared to genomics, transcriptomics or proteomics, given its ability to characterize the molecular phenotype (147). This aspect makes it possible to significantly improve clinical approach to pituitary disorders in a personalized manner, for example in the diagnosis of MRI negative ACTH-secreting PA or gonadotropin-secreting PA. Likewise, identification of new metabolomic biomarkers in GH-secreting PA would considerably alleviate the prognosis of acromegaly and, potentially, predict therapeutic response. Characterization of specific metabolic pathways underlying PA including their functional alteration would bring new data into understanding their pathogenesis, thus making it possible to identify new therapeutic targets.

Metabolomics seems to be a promising tool in the future in neurosurgery, given that LC/MS and nanostructure imaging mass spectrometry (NIMS) have been able to identify brain region mapping on the animal model (148) and that first steps toward clinical application of MALDI MSI were initiated (63). Nonetheless, further studies are warranted to confirm preliminary results and deepen knowledge in the field.

## Conclusion

Implementation of ultra high performance metabolomics analysis techniques in the study of PA will significantly improve diagnosis and, potentially, the therapeutic approach, by identifying highly specific disease biomarkers in addition to novel molecular pathogenic mechanisms. Ultra high mass resolution MALDI-MSI emerges as a helpful clinical tool in the neurosurgical treatment of pituitary tumors. Therefore, metabolomics appears to be a science with a promising prospect in the sphere of PA, and a starting point in pituitary care.

## Author Contributions

OP collected data, drafted the manuscript and wrote the review. BG contributed to the data collection. CG conceived the study design and wrote the review.

### Conflict of Interest Statement

The authors declare that the research was conducted in the absence of any commercial or financial relationships that could be construed as a potential conflict of interest.
